# Modulation of Innate Antigen-Presenting Cell Function by Pre-patent Schistosome Infection

**DOI:** 10.1371/journal.pntd.0002136

**Published:** 2013-03-21

**Authors:** Christine E. Ferragine, Colleen D. Walls, Stephen J. Davies

**Affiliations:** Department of Microbiology and Immunology, Uniformed Services University of the Health Sciences, Bethesda, Maryland, United States of America; Cambridge University, United Kingdom

## Abstract

Schistosomes are intravascular helminths that infect over 200 million people worldwide. Deposition of eggs by adult schistosomes stimulates Th2 responses to egg antigens and induces granulomatous pathology that is a hallmark of schistosome infection. Paradoxically, schistosomes require host immune function for their development and reproduction and for egress of parasite eggs from the host. To identify potential mechanisms by which immune cells might influence parasite development prior to the onset of egg production, we assessed immune function in mice infected with developing schistosomes. We found that pre-patent schistosome infection is associated with a loss of T cell responsiveness to other antigens and is due to a diminution in the ability of innate antigen-presenting cells to stimulate T cells. Diminution of stimulatory capacity by schistosome worms specifically affected CD11b^+^ cells and did not require concomitant adaptive responses. We could not find evidence for production of a diffusible inhibitor of T cells by innate cells from infected mice. Rather, inhibition of T cell responsiveness by accessory cells required cell contact and only occurred when cells from infected mice outnumbered competent APCs by more than 3∶1. Finally, we show that loss of T cell stimulatory capacity may in part be due to suppression of IL-12 expression during pre-patent schistosome infection. Modulation of CD4^+^ T cell and APC function may be an aspect of host immune exploitation by schistosomes, as both cell types influence parasite development during pre-patent schistosome infection.

## Author Summary

The disease schistosomiasis is caused by a parasitic blood fluke found mainly in the tropics and subtropics and affects over 200 million people worldwide. Using mice to model human schistosome infection, our previous studies showed that schistosome development in the infected host is linked to host immune function, such that parasite development is impaired in hosts with immunological deficiencies. CD4^+^ T cells and cells of the monocyte/macrophage lineage are two types of immune cells that are involved in modulating schistosome development. In this study, we examined immune function in mice infected with developing schistosomes, to gain insights into how immune cells might influence parasite development. We found evidence of broad-spectrum suppression of CD4^+^ T cell responses during early schistosome infection. We also show that the loss of T cell responsiveness is due to impairment of T cell stimulation by CD11b^+^ cells. These findings suggest that exploitation of CD4^+^ T cells and monocytes/macrophages by schistosomes may involve parasite modification of the function of these cells.

## Introduction

Schistosomes are intravascular helminths affecting approximately 200 million people throughout the tropics and subtropics [1], [2], with more than 90% of cases occurring in sub-Saharan Africa [3]. Upon infection, a variety of host responses are induced. Exposure of antigen-presenting cells (APCs) in the skin to invading cercariae stimulates APC migration to the draining lymph nodes and induction of transient parasite-specific T helper (Th) 2 responses [4]. While mononuclear cells and neutrophils infiltrate the skin in response to the penetration of cercariae [5], evidence suggests that schistosomula in the skin elicit an immuno-modulatory environment, by secreting an anti-inflammatory protein [6] and inducing the production of the eicosanoid, prostaglandin E-2 (PGE_2_), which suppresses T cell proliferation by an interleukin (IL-) 10-dependent mechanism [7]. Onward parasite migration into the circulatory system induces a mixed systemic response, with evidence of both Th2 [8] and modest Th1 induction [9]. The former is necessary and sufficient to induce production of antigen-specific IgE and cause sensitization of basophils to produce further IL-4 in response to worm antigens [8]. At approximately 5–6 weeks post infection, parasite egg production commences and stimulates a robust, predominantly Th2 response [10] [11], while prior responses to worm antigens are down-regulated [9].

Schistosomes can persist in the host for an average of 5–10 years [12], evading immune destruction to establish long term, chronic infections [1]. Chronic infections in general [13], [14] and helminth infections in particular [15], [16] are associated with the induction of an immunologically hyporesponsive state where either innate or adaptive immune functions, or both, are modulated [17], [18], [19]. Examples of modulation in innate immune function by helminths have been documented previously. For instance, protease inhibitors found in helminth excretory-secretory (ES) products, such as cystatins, inhibit cysteine proteases required for APC antigen processing and presentation [20]. Helminth cystatins also elicit the production of the immunosuppressive cytokine IL-10, reducing expression of co-stimulatory molecules on APCs and inhibiting T cell proliferation [21]. Other secreted helminth products, such as ES-62 and schistosome-expressed glycoconjugates, suppress macrophage IL-12 production [22] and induce suppressor macrophages [23], respectively. Finally, schistosome lyso-phosphatidylserine (lyso-PS), another immunomodulatory glycoconjugate, stimulates dendritic cells (DC) to induce IL-10 secreting regulatory T cells (Treg), leading to regulation of the T cell response [24].

Modulation of the antigen-presenting capabilities of innate cells by helminths may be implicated in the modulation of adaptive immune function and the induction of regulatory Treg responses later in infection [25]. The regulatory cytokine IL-10 has been shown to inhibit T cell activation by downregulating MHCII and B7 expression on APCs [26], thus leading to decreased T cell responses. Patients chronically infected with *Schistosoma haematobium* [27], [28] and *S. mansoni* [29] exhibit hyporesponsiveness in their T lymphocyte populations, characterized by reduced proliferation and interferon (IFN-) γ production in response to parasite antigen. Not only is the parasite-specific T cell response down modulated [30], but so is the overall T cell response that is elicited by polyclonal stimulation [31]. There is speculation that the immune modulation brought about by helminth infection may be an adaptive mechanism used by worms for their own advantage, as preventing destructive immunopathology prolongs survival of both parasite and host [17], [32]. Consistent with this hypothesis, excessive Th1 [33] and Th2 responses [34] to egg antigens are both detrimental to the host and are subject to regulation by IL-10 [35]. IL-10-mediated suppression of T helper responses has been shown to reduce the pathology associated with schistosomiasis by regulating the development of severe egg-induced pathology [34].

Using a mouse model of *S. mansoni* infection, we previously showed that worm development and maturation requires host immune function [36]. In immunodeficient mice that lack T and B cells, schistosome growth and sexual maturation are impaired, and adoptive transfer of CD4^+^ T cells is sufficient to restore worm development [37]. Thus CD4^+^ T cells might not only benefit schistosomes by modulating pathology and prolonging host survival, but might also contribute to providing an environment conducive to parasite growth and development. However, the mechanism underpinning this latter process is still not clear. To gain insights into how CD4^+^ T cells contribute to the growth and development of schistosomes, we sought to delineate the interactions that occur between schistosomes and CD4^+^ T cells during the pre-patent stage of infection. We show that the response to pre-patent schistosome infection is associated with a loss of T cell responsiveness to other antigens, and that loss of T cell responsiveness is due to a specific diminution in the ability of innate APCs to stimulate T cells. Modulation of CD4^+^ T cell and innate APC function during pre-patent schistosome infection may represent one aspect of the exploitation of these cell populations by schistosomes during the establishment of infection [38].

## Materials and Methods

### Ethics statement

All animal studies were performed in strict accordance with the recommendations of the Office of Laboratory Animal Welfare at the National Institutes of Health and the USUHS Institutional Animal Care and Use Committee. All animal experiments were performed in accordance with protocols previously approved by the USUHS Institutional Animal Care and Use Committee.

### Mice

C57BL/6 wild type mice were purchased from National Cancer Institute (Frederick, MD). Recombination activating gene (RAG-) 1^−/−^ mice and OT-II/RAG-1^−/−^ mice [39] on a C57BL/6 background were purchased from Jackson Laboratory (Bar Harbor, ME) and the Taconic/NIAID Emerging Models Program and bred in house to obtain sufficient numbers of animals for experiments.

### *Schistosoma mansoni* infection

Infected *Biomphalaria glabrata* snails were provided by Dr. Fred Lewis (BRI, Rockville, MD) and maintained in house to produce mixed male and female cercariae of the Puerto Rican strain of *Schistosoma mansoni*. Mice of 4–6 weeks of age were infected percutaneously by tail immersion in water containing approximately 150 *S. mansoni* cercariae for 45 minutes [40], [41]. For all experiments mice were sacrificed at 4 weeks post infection and included 5 mice per group, with each mouse analyzed individually. Schistosome worm antigen preparation (SWAP) was prepared from adult *S. mansoni* worms perfused from the portal veins of infected mice. Worms were homogenized in phosphate buffered saline (PBS) on ice. Homogenates were centrifuged at 16,100 g for 30 minutes at 4°C to remove insoluble material and the remaining supernatant was filter sterilized and stored at −80°C, after determination of protein concentration using Bradford Assay.

### Cell isolation and culture

Single cell leukocyte suspensions were prepared by forcing splenic, hepatic or lymph node tissue through 70 µm nylon cell strainers. Hepatocytes were removed from liver using 30% Percoll density gradient centrifugation. Erythrocytes were lysed using ACK lysing buffer (Quality Biological, Inc.) CD4^+^ T cells, CD11c^+^, or CD11b^+^ cells were isolated by magnetic cell separation after incubation with anti-CD4, anti-CD11c, or anti-CD11b microbeads, respectively, using MACS cell separation columns (Miltenyi Biotech) according to manufacturer's protocols. Cells were cultured at 2×10^6^ cells/ml in RPMI (Gibco)/10% fetal bovine serum/20 mM glutamine/0.1 mM MEM non-essential amino acids/1 mM sodium pyruvate/10 mM HEPES buffer solution/100 U/ml penicillin/100 µg streptomycin/50 µM 2-mercaptoethanol. APCs were co-cultured with CD4^+^ T cells at a ratio of 1 APC:10 CD4^+^ T cells. Cell cultures were stimulated with SWAP at 50 µg/ml [42], OVA_323–339_ peptide at 2.5 µg/ml (Anaspec), anti-CD3 monoclonal antibody (clone 145-2C11, BD Bioscience) at 1 µg/ml, IL-2 at 5 ng/ml, or rIL-12 at 100 ng/ml (Peprotech) for 72 hours at 37°C and 5% CO_2_. Other additives were used at the following concentrations: N^ω^-hydroxy-nor-arginine (nor-NOHA) at 500 µg/ml (Calbiochem), L-N^G^-monomethyl arginine citrate (L-NMMA) at 500 µg/ml (Calbiochem), blocking anti-IL-10R monoclonal antibody (clone 1B1.3a, kindly provided by Dr. Yasmine Belkaid) at 10 µg/ml, blocking anti-CD274 (PD-L1) monoclonal antibody (clone MIH5, eBioscience) at 500 ng/ml, or anti-TGF-β1, 2, 3 monoclonal antibody (clone 1D11, R&D Systems)at 1.5 µg/ml,.

### Quantification of cytokine production

Cells were cultured as described and supernatants were collected and stored at −80°C. Concentrations of IFN-γ, IL-10, or IL-4 was measured per manufacturer's protocols using BD Opt EIA ELISA sets (BD Bioscience) and analyzed at 450 nm with λ correction of 570 nm using a Spectramax M2 plate reader (Molecular Devices).

### Real time PCR

Splenic or hepatic tissue was homogenized in RNAzol (Tel-Test, Inc.) and RNA isolated according to manufacturer's directions. High-purity RNA was then isolated with RNeasy mini columns, with on-column DNase digestion using RNase-Free DNase Set (Qiagen). RNA was analyzed for purity and concentration using a NanoDrop ND-1000 Spectrophotometer (Wilmington, DE) and 1 µg RNA used to make cDNA with High Capacity RNA to cDNA kit (Applied Biosystems) and M.J. Research DNA Engine DYAD thermal cycler (Bio-Rad). Real time PCR was performed using Taqman Gene Expression assays for IL-12p35 and GAPDH (Applied Biosystems), per manufacturer's protocols, using M.J. Research Chromo4 PCR cycler (Bio-Rad) and Opticon Monitor 2 Analysis Software (M.J. Research) to quantitate C_T_ values. Fold change was calculated using the comparative C_T_ method with GAPDH as the endogenous control [43].

### Cell surface molecule expression

For analysis of APC populations, cells isolated from spleen and liver of wild type mice were stained with Live-Dead Aqua fixable dead cell stain (Invitrogen) and the following monoclonal antibodies: phycoerythrin- (PE-) conjugated anti-B220 (clone RA3-6B2), allophycocyanin-conjugated anti-CD11c (clone HL3), PE- or peridinin chlorophyll protein- (PerCP-) Cy5.5-conjugated anti-CD11b (clone M1/70), Fluorescein isothiocyanate-conjugated anti-CD86 (clone GL1), PerCP-Cy5.5-conjugated anti-Ly-6C (clone AL-21) (all from BD Biosciences), and Alexa Fluor 700-conjugated anti-MHC II (clone M5/114.15.2) (eBioscience). Cells were gated on forward scatter (FSC-H/FSC-A) to exclude doublets, on FSC-H and side scatter (SSC-H) to exclude granulocytes, and on Live-Dead Aqua-negative events to exclude dead cells. Percentages of CD11b^+^ and CD11c^+^ populations were determined and used to calculate total number of each cell type per organ. All samples were analyzed on a LSR II Optical Bench Flow Cytometer using FACSDiva (BD Biosciences) and Winlist software (Verity Software House).

### Proliferation assay

Splenocytes or isolated CD4^+^ T cells from OT-II/RAG-1^−/−^ mice were stained with carboxyfluorescein diacetate succinimidyl ester (CFDA-SE, or CFSE; Invitrogen), according to manufacturer's recommendations using Cell Trace CFSE cell proliferation kit (Invitrogen). CFSE stained cells were co-cultured as described above with either unfractionated splenocytes from RAG-1^−/−^ mice, or with CD11c^+^ or CD11b^+^ cells isolated by magnetic cell sorting, as described above. Cells from 72 hour co-cultures were collected and stained with APC-Cy7-conjugated anti-CD4 (cloneGK1.5,BD Bioscience), and Live-Dead Aqua. After gating on live CD4^+^ T cells, CFSE staining was analyzed by flow cytometry and used to determine the CD4^+^ T cell proliferation index using Modfit LT Proliferation Wizard (Verity Software House).

### Transwell experiments

In the lower chamber of a 24-well Transwell plate (Corning), CD4^+^ T cells isolated from OT-II/RAG-1^−/−^ mice and stained with CFSE were co-cultured with splenocytes from non-infected RAG-1^−/−^ mice, at a ratio of 10∶1 in the presence of OVA peptide. Equal numbers of splenocytes from non-infected or infected RAG-1^−/−^ mice were added to the upper chamber. After culture for 72 hours, CD4^+^ T cell proliferation was analyzed as described above. Transwell cultures with no OVA stimulation were included as controls.

### APC competition assay

CD4^+^ T cells isolated from OT-II/RAG-1^−/−^ mice were co-cultured with splenic antigen presenting cells from RAG-1^−/−^ mice at a ratio of 1 APC:10 T cells as described above. The APC population consisted of splenocytes from non-infected and infected mice mixed in varying proportions. Cytokine production and CD4^+^ T cell proliferation were analyzed as described above.

### Statistical analysis

Due to unequal variances among some of the experimental groups analyzed, non parametric tests were used to test for significant differences between groups. For comparisons between two groups, Mann-Whitney test was used and for comparisons between more than two groups, Kruskal-Wallis test was used followed by Dunn's multiple comparison tests. GraphPad Prism Software Version 5 (GraphPad Software Inc., San Diego, CA) was used to perform all statistical analyses. *P* values of 0.05 or less were considered significant. All experiments were repeated at least twice with 5 animals per group.

## Results

### Pre-patent schistosome infection impairs IFN-γ production and proliferation of CD4^+^ T cells in response to polyclonal stimulation

**Figure 1:**
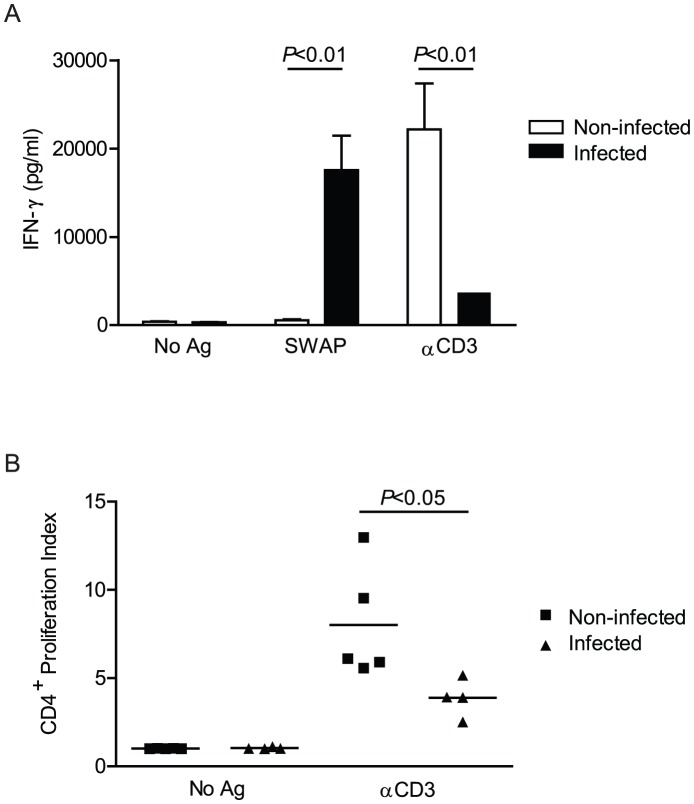
Pre-patent schistosome infection impairs T cell production of IFN-γ and proliferation of CD4^+^ T cells in response to polyclonal stimulation. A, Splenocytes isolated from 4 week *S. mansoni*-infected or non-infected wild type mice were cultured *in vitro* for 72 hours without antigen, with SWAP or with anti-CD3 antibody. The concentration of IFN-γ was measured from cell culture supernatant by ELISA. Data shown are mean +/− SEM. B, Splenocytes isolated from 4 week-infected or non-infected wild type mice were stained with CFSE and cultured for 72 hrs without antigen or with αCD3 antibody. Cells were recovered from culture, stained for surface markers, then CFSE dilution due to cell division was analyzed by flow cytometry and proliferation index calculated. Horizontal bars represent mean value of 5 independent mice. Data are representative of 3 independent experiments. No Ag, no antigen; SWAP, soluble worm antigen preparation; αCD3, anti-CD3 antibody.

To determine the effect of pre-patent schistosome infection on the ability of CD4^+^ T cells to produce cytokines and proliferate, we isolated splenic leukocytes from 4 week *S. mansoni*-infected wild type mice and assessed their ability to produce IFN-γ in response to specific antigens and polyclonal stimulation. Cells from infected mice exhibited robust production of IFN-γ in response to soluble worm antigen preparation (SWAP), whereas cells from non-infected mice produced almost no IFN-γ. However, in response to polyclonal T cell stimulation with anti-CD3 antibody, cells from infected mice were significantly impaired in their ability to produce IFN-γ as compared to cells from non-infected mice (Fig. 1A). Furthermore, proliferation of CD4^+^ T cells from the spleens of infected wild type mice was significantly reduced in response to polyclonal stimulation (Fig. 1B).These data suggest that pre-patent schistosome infection reduced the ability of T cells to produce IFN-γ and specifically altered CD4^+^ T cell proliferation in response to polyclonal T cell stimulation.

### Pre-patent schistosome infection impairs CD4^+^ T cell responses to non-schistosome antigens

**Figure 2:**
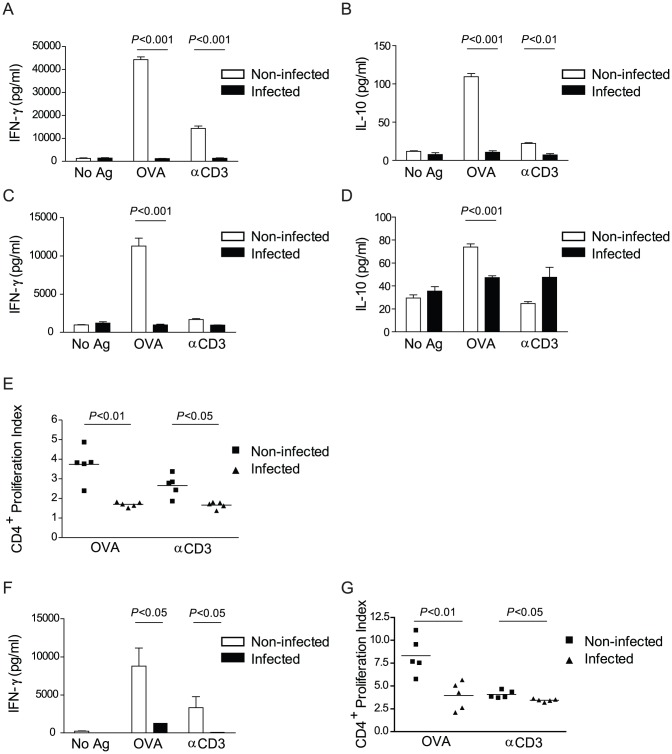
Pre-patent schistosome infection impairs CD4^+^ T cell responses to non-schistosome antigens. A–D, Splenocytes (A–B) or hepatic leukocytes (C–D) isolated from 4 week *S. mansoni*-infected or non-infected OT-II/RAG-1^−/−^ mice were cultured *in vitro* for 72 hours without antigen, or in the presence of OVA peptide or anti-CD3 antibody. The concentration of IFN-γ (A,C) or IL-10 (B,D) in the cell culture supernatant was measured by ELISA. Data shown are mean +/− SEM. E, Splenocytes were isolated from 4 week-infected or non-infected OT-II/RAG-1^−/−^ mice, stained with CFSE, and cultured for 72 hrs with OVA peptide or anti-CD3 stimulation. Cells were recovered from culture, stained for surface markers, then CFSE dilution due to cell division was analyzed by flow cytometry and proliferation index calculated. Horizontal bars represent mean value of 5 independent mice. F–G, Splenocytes were isolated from 4 week *S. mansoni*-infected or non-infected RAG-1^−/−^ mice; CD4^+^ T cells were isolated from non-infected OT-II/RAG-1^−/−^ mice and stained with CFSE. Cells were then co-cultured for 72 hours without antigen or in the presence of OVA peptide or anti-CD3 antibody. The concentration of IFN-γ in cell culture supernatants was measured by ELISA (F) and CD4^+^ T cell proliferation was analyzed by flow cytometry (G). Data are representative of at least 3 independent experiments. No Ag, no antigen; OVA, OVA peptide; αCD3, anti-CD3.

While pre-patent schistosome infection stimulates the expansion of antigen-specific CD4^+^ T cells that proliferate and produce cytokines in response to schistosome antigens *in vitro* [8], the frequency of schistosome-specific T cells relative to T cells with other specificities is suspected to remain relatively low. In contrast, anti-CD3 antibody is an artificial polyclonal stimulus that stimulates all T cells, regardless of specificity. We therefore hypothesized that the reduced responsiveness to anti-CD3 observed in infected mice was due to loss of responsiveness in the T cell population in general, regardless of TCR specificity. To test the effect of infection on T cells with specificity for non-schistosome antigens, we infected TCR-transgenic OT-II/RAG-1^−/−^ mice that only possess OVA-specific CD4^+^ T cells. When splenocytes (Fig. 2A, 2B) and hepatic leukocytes (Fig. 2C, 2D) were isolated from non-infected OT-II/RAG-1^−/−^ mice, stimulation with OVA peptide induced robust production of IFN-γ (Fig. 2A, 2C) and IL-10 (Fig. 2B, 2D). Stimulation with anti-CD3 antibody also stimulated IFN-γ and IL-10 production by splenocytes (Fig. 2A, 2B), but less robustly than OVA peptide, perhaps indicating that, when all the T cells share the same specificity, cognate stimulation with peptide is a more efficient T cell stimulus than TCR ligation with antibody. In contrast, when splenocytes and hepatic leukocytes isolated from infected OT-II/RAG-1^−/−^ mice were stimulated with OVA peptide, the production of IFN-γ and IL-10 was significantly less when compared to cells from non-infected mice and was comparable to that observed without stimulation (Fig. 2A–D). Likewise, production of IFN-γ and IL-10 in response to anti-CD3 stimulation was also significantly reduced in cultures of splenocytes from infected OT-II/RAG-1^−/−^ mice (Fig. 2A, 2B). Additionally, CD4^+^ T cells in splenocyte cultures from infected OT-II/RAG-1^−/−^ mice proliferated significantly less in response to OVA peptide or anti-CD3 when compared to cells from non-infected mice (Fig. 2E). To test whether schistosome infection directly altered the ability of OT-II T cells to produce cytokines and proliferate, we isolated splenocytes from infected or non-infected RAG-1 ^−/−^ mice and used them as APCs to stimulate CD4^+^ T cells isolated from non-infected OT-II/RAG-1^−/−^ mice. When splenocytes from infected RAG-1 ^−/−^ mice were used as APCs, production of IFN-γ in response to either OVA peptide or anti-CD3 was significantly reduced compared to co-cultures containing splenocytes from non-infected RAG-1^−/−^ mice (Fig. 2F). Likewise, CD4^+^ T cell proliferation in response to OVA peptide and anti-CD3 was also significantly reduced in co-cultures with splenocytes from infected mice (Fig. 2G). These data suggest that alteration of CD4^+^ T cell responses by pre-patent schistosome infection is due to alteration of the ability of accessory cells to stimulate T cells rather than a direct effect on CD4^+^ T cells themselves, i.e. that T cell hyporesponsiveness does not occur as a result of some infection-induced cell-intrinsic change in T cell function. These data are consistent with our previous findings that schistosome infection did not alter the responsiveness of OT-II T cells in OT-II/RAG-1^−/−^mice [38]. Furthermore, these data demonstrate that pre-patent schistosome infection directly modulates the APC function of innate accessory cells, independent of adaptive responses, as the accessory cells used in these experiments were obtained from RAG-1^−/−^ mice.

### Splenic and hepatic CD11c^+^ and CD11b^+^ populations are maintained during pre-patent schistosome infection

**Figure 3:**
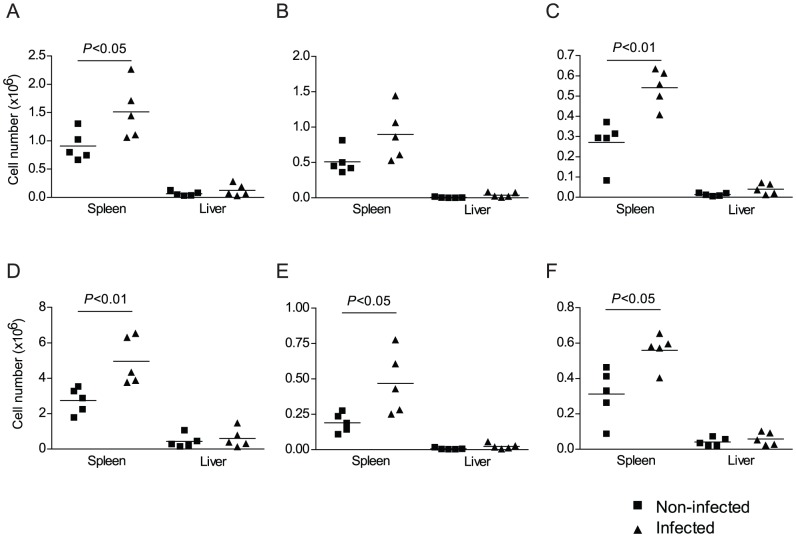
Numbers of splenic and hepatic CD11c^+^ and CD11b^+^ cells during pre-patent schistosome infection. A, Flow cytometric analysis of cells isolated from the spleen or liver of 4 week *S. mansoni* infected or non-infected mice. Cells were gated and the absolute number of cells of each phenotype was calculated as described in Methods. Total numbers of CD11c^+^ (A), CD11c^+^ MHC class II^+^ (B) or CD11c^+^ CD86 (C) cells per organ. Total numbers of CD11b^+^ (D), CD11b^+^ MHC class II^+^ (E) or CD11c^+^ CD86^+^ (F) cells per organ. Horizontal bars represent the mean of 5 independent mice.

To examine whether pre-patent infection induced changes in the number and phenotype of innate APCs, splenic and hepatic populations of CD11c^+^ dendritic cells and CD11b^+^ mononuclear cells in infected and non-infected wild type mice were compared by flow cytometry. There was a significant increase in the total number of CD11c^+^ cells in the spleens of infected mice when compared to non-infected controls (Fig. 3A). Furthermore, the total numbers of CD11c^+^ MHC II^+^ (Fig. 3B) and CD11c^+^ CD86^+^ cells (Fig. 3C) in the spleen were also increased by infection, although only the latter attained statistical significance. For CD11b^+^ mononuclear cells, the total number of cells in the spleen was significantly increased by infection (Fig. 3D), as were the numbers of CD11b^+^ MHC II^+^ (Fig. 3E) and CD11b^+^ CD86 (Fig. 3F). Although the total numbers of CD11c^+^ dendritic cells and CD11b^+^ mononuclear cells in the liver were lower than in the spleen, a similar trend towards higher cell numbers was observed in infected mice (Fig. 3A–F). These data suggest that both CD11c^+^ and CD11b^+^ APC populations increase, or are at least maintained, in response to schistosome infection and that the alteration of T cell responsiveness in splenocyte and hepatic leukocyte cultures is not due to a reduction in APC numbers.

### Pre-patent schistosome infection specifically impairs the ability of CD11b^+^ cells to stimulate CD4^+^ cells

**Figure 4:**
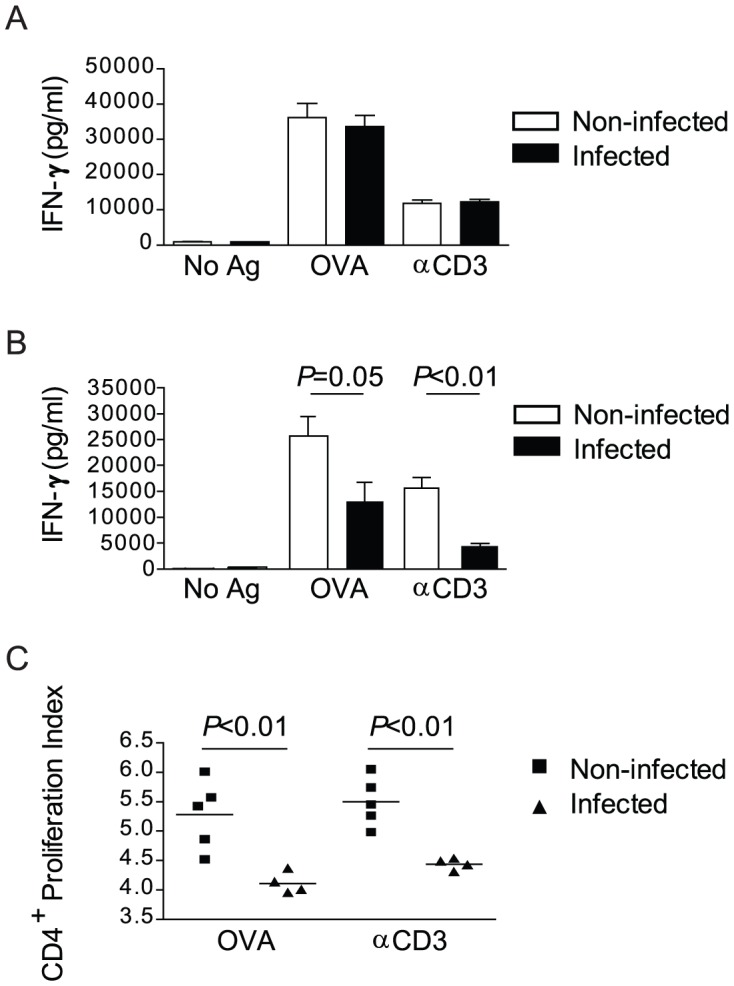
Pre-patent schistosome infection impairs the ability of CD11b^+^ cells to stimulate CD4^+^ cells. A, CD11c^+^ dendritic cells isolated from the spleen of 4 week *S. mansoni*-infected or non-infected wild type mice and CD4^+^ T cells isolated from non-infected OT-II/RAG-1^−/−^ mice were co-cultured for 72 hours without antigen, with OVA peptide or with anti-CD3 antibody. The concentration of IFN-γ in the cell culture supernatants was measured by ELISA. B, CD11b^+^ cells were isolated from the spleens of 4 week *S. mansoni*-infected or non-infected OT-II/RAG-1^−/−^ mice; CD4^+^ T cells were isolated from non-infected OT-II/RAG-1^−/−^ mice, and stained with CFSE. Cells were then co-cultured for 72 hours without antigen, with OVA peptide or with anti-CD3 antibody. The concentration of IFN-γ in the cell culture supernatants was measured by ELISA. Data shown are mean +/− SEM. C, CD4^+^ T cell proliferation in the co-cultures stimulated with OVA peptide or anti-CD3 antibody described in (B) was analyzed by flow cytometry and proliferation index calculated. Horizontal bars represent mean of 5 independent mice. No Ag, no antigen; OVA, OVA peptide; αCD3, anti-CD3.

To determine whether the loss in T cell stimulatory capacity was associated specifically with either CD11c^+^ or CD11b^+^ cells, these populations were isolated from the spleens of infected and non-infected mice and their ability to stimulate OT-II CD4^+^ T cells *in vitro* was examined. OVA peptide or anti-CD3 stimulation of OT-II T cell/CD11c^+^ cell co-cultures induced similar levels of IFN-γ production, regardless of whether the CD11c^+^ cells used as APCs originated from infected or non-infected mice (Fig. 4A). However, when CD11b^+^ cells were used as APCs, a significant reduction in the production of IFN-γ in response to anti-CD3 was observed when the CD11b^+^ cells were isolated from infected mice (Fig. 4B). Likewise, the production of IFN-γ in response to OVA peptide in OT-II T cell/CD11b^+^ cell co-cultures was diminished when the CD11b^+^ cells were obtained from infected mice, although this result narrowly avoided attaining statistical significance (*P* = 0.0501; Fig. 4B). Finally, CD11b^+^ cells from infected mice stimulated significantly less proliferation of OT-II CD4^+^ T cells than CD11b^+^ cells from non-infected mice, whether the cells were stimulated with OVA peptide or anti-CD3 stimulation (Fig. 4C). These data suggest that pre-patent schistosome infection specifically impairs the ability of CD11b^+^ accessory cells to stimulate T cells, but does not alter the T cell stimulatory capacity of CD11c^+^ dendritic cells.

### Two populations of CD11b^+^ cells in RAG-1^−/−^ mice

**Figure 5:**
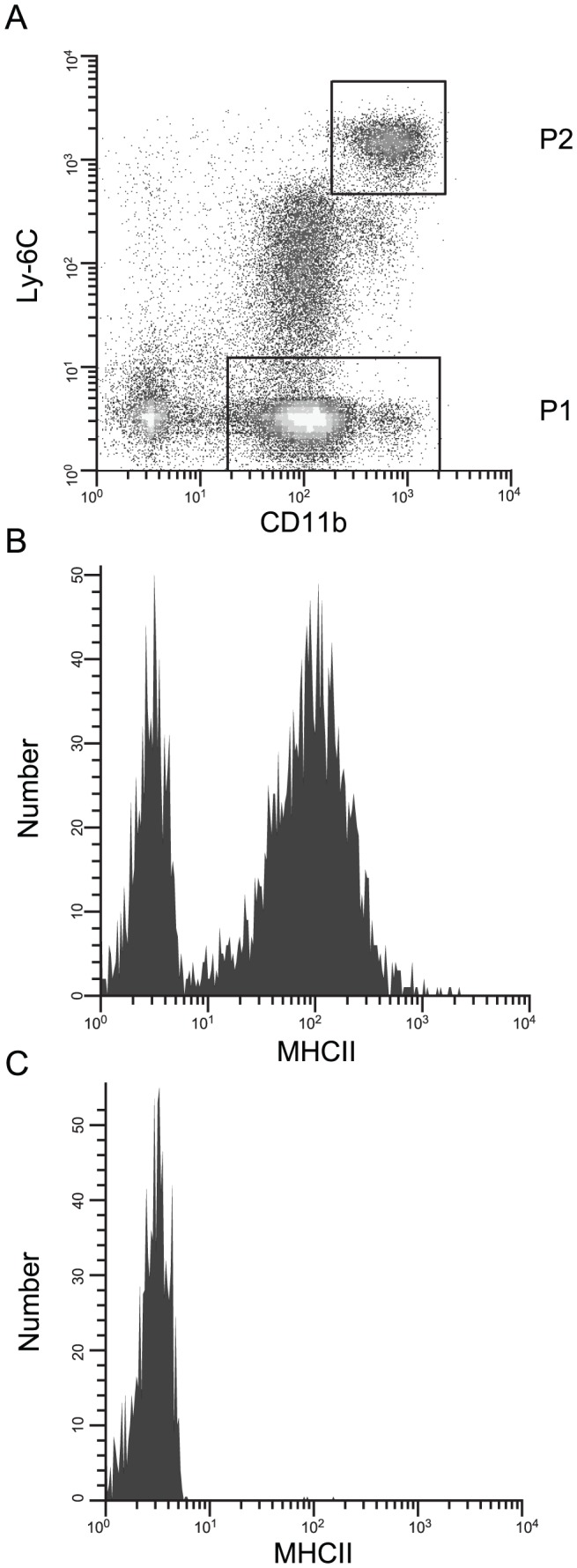
CD11b^+^ cell populations in RAG^−/−^ mice. A, Flow cytometric analysis of CD11b and Ly-6C expression by splenocytes from RAG-1^−/−^ mice. Gates encompassing the two main cell populations are labeled P1 and P2. B, MHC class II expression by cells contained within gate P1. C, MHC class II expression by cells contained within gate P2.

To further characterize the CD11b^+^ cell populations present in RAG-1^−/−^ mice, expression of CD11b and Ly-6C by splenocytes from these animals was analyzed by flow cytometry. Two main cell populations could be discerned (Fig. 5A) – one that mostly expressed low levels of CD11b and no Ly-6C (designated P1 in Fig. 5A), and one that expressed high levels of both CD11b and Ly-6C (designated P2 in Fig. 5A). Analysis of MHC II expression revealed that the majority of P1 cells express MHC II (Fig. 5B), while P2 cells did not express MHC II (Fig. 5C). This pattern is consistent with P1 representing more mature macrophages/mononuclear phagocytes that have acquired antigen-presenting capabilities, and P2 representing an earlier monocyte developmental state. The morphology of cells from these two populations when isolated by flow cytometry and examined by cytospin was consistent with this conclusion (data not shown). We also noted a less well-defined population of cells with variably intermediate CD11b and Ly-6C expression levels between P1 and P2, which may represent cells maturing from the P2 phenotype to the P1 phenotype. This overall staining pattern for CD11b, Ly-6C and MHC II did not differ significantly between infected and non-infected RAG-1^−/−^ mice (not shown).

### Impairment of T cell stimulation by pre-patent schistosome infection is independent of arginase, NOS, IL-10, PD-L1 and TGF-β

**Figure 6:**
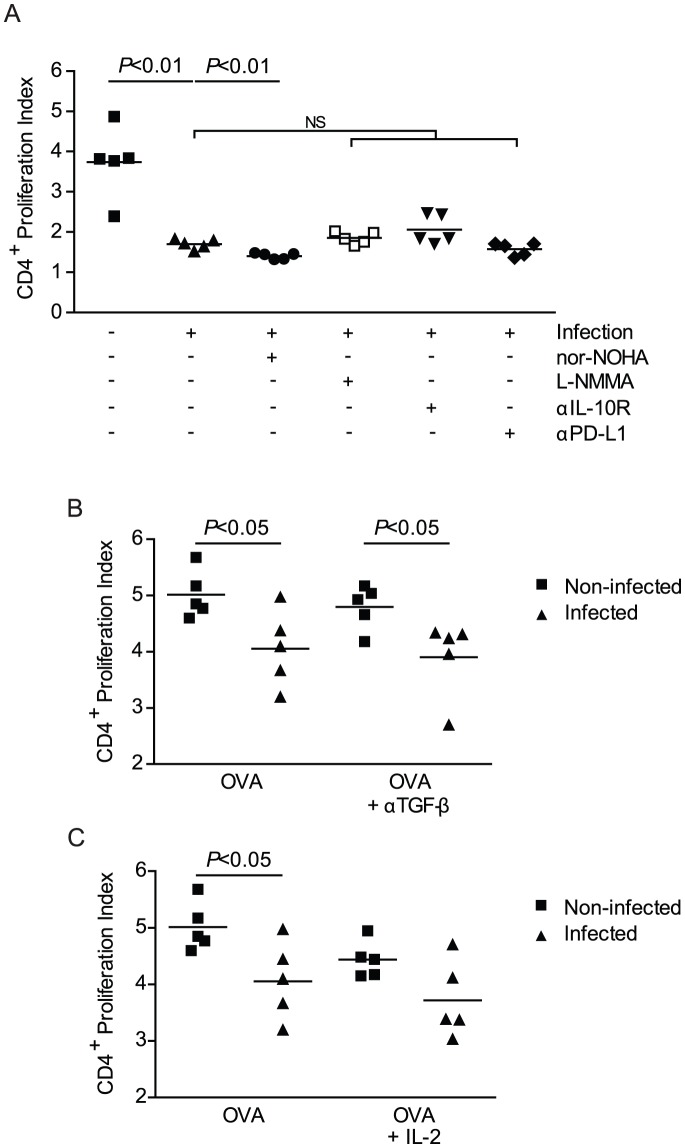
Impairment of T cell stimulation by pre-patent schistosome infection is independent of arginase, NOS, IL-10, PD-L1 and TGF-β. A, Splenocytes isolated from 4 week *S. mansoni*-infected or non-infected RAG-1^−/−^ mice co-cultured with CFSE-stained CD4^+^ T cells isolated from non-infected OT-II/RAG-1^−/−^ mice for 72 hrs with OVA peptide stimulation in the presence of Nor-NOHA, L-NMMA, anti-IL-10R or anti-PD-L1. Cells recovered from culture were stained for surface markers then analyzed for CD4^+^ T cell proliferation by flow cytometry. Proliferation Index was calculated. Horizontal bars represent mean of 5 independent mice. B, In a replicate, independent experiment, cells were isolated as in (A) and cultured in the presence of OVA peptide stimulation with anti-TGF-β1, β2, β3 antibody (B) or recombinant IL-2 (C) and. CD4^+^ T cell proliferation was analyzed by flow cytometry. Horizontal bars represent mean of 5 independent mice. Data are representative of at least 3 independent experiments. Nor-NOHA, N^ω^-hydroxy-nor-Arginine; L-NMMA, L-N^G^-monomethyl Arginine citrate; αIL-10R, anti-IL-10 receptor monoclonal antibody; αPD-L1, anti-PD-L1 monoclonal antibody; αTGF-β, anti-TGF-β1, β2, β3 monoclonal antibody.

We hypothesized that the impairment in CD4^+^ T cell stimulation associated with CD11b^+^ cells could result from the induction of a suppressor mechanism in CD11b^+^ cells, as suppressor macrophages that induce T cell anergy have previously been described in mice harboring patent schistosome infections [23], [44]. In these studies, macrophages were found to suppress T cell proliferation through expression of IL-10 or the B7 family member programmed death ligand-1 (PD-L1), also known as B7-H1. Furthermore, other suppressor mechanisms requiring arginase-1 or nitric oxide synthase have been implicated in the suppression of T cell responses by myeloid derived suppressor cells (MDSCs) [45]. To investigate whether these mechanisms mediate T cell suppression induced by pre-patent schistosome infection, we tested whether inhibitors of these pathways could restore OVA-induced OT-II T cell proliferation in co-cultures where splenocytes from infected RAG-1 ^−/−^ mice were used as APCs. Nor-NOHA was used to specifically inhibit arginase and L-NMMA was used to inhibit nitric oxide synthase (NOS), both enzymes previously implicated in the inhibition of T cell proliferation by MDSCs [46]. To determine whether the infection-induced impairment in T cell proliferation is mediated by the regulatory cytokine IL-10, IL-10 signaling was inhibited in co-cultures by the addition of anti-IL-10 receptor monoclonal antibody. PD-L1 was inhibited using an anti-PD-L1 monoclonal antibody. None of these treatments restored proliferation to levels seen with non-infected APCs. When L-NMMA, anti-IL-10R or anti-PD-L1 were included in co-cultures with splenocytes from infected RAG-1^−/−^ mice, OT-II T cell proliferation was not significantly different from that observed in control cultures without inhibitor, and was still significantly less than the proliferation observed in co-cultures with splenocytes from non-infected mice (Fig. 6A). In cultures containing Nor-NOHA, proliferation of OT-II T cells was reduced even further when compared to control cultures lacking inhibitor (Fig. 6A). Consistent with these results, no restoration of proliferation was observed when these inhibitors were added to cultures containing un-fractionated splenocytes from infected and non-infected OT-II/RAG-1^−/−^ mice (data not shown). Addition of neutralizing anti-transforming growth factor- β1, β2, β3 (anti-TGF-β1, β2, β3) monoclonal antibody to co-cultures did not restore OT-II T cell proliferation in the presence of splenocytes from infected RAG-1^−/−^ mice (Fig. 6B). These results suggest the impaired stimulation of CD4^+^ T cells is independent of NOS, IL-10, PD-L1 and TGF-β, while arginase activity may support T cell proliferation. Finally, we tested whether addition of exogenous IL-2 could restore OT-II T cell proliferation in the presence of splenocytes from infected RAG-1^−/−^ mice, as exogenous IL-2 has previously been shown to restore proliferation in anergic T cells [14], [47], [48]. However, the addition of IL-2 to co-cultures did not lead to restoration of T cell proliferation in the presence of infected RAG-1^−/−^ splenocytes (Fig. 6C). Indeed, the addition of exogenous IL-2 to co-cultures with non-infected RAG-1^−/−^ splenocytes inhibited proliferation, such that the level of proliferation was no longer significantly different than in co-cultures containing infected RAG-1^−/−^ splenocytes (Fig. 6C). Taken together, these data suggest that the loss of T cell proliferation in the presence of splenocytes from infected RAG-1^−/−^ mice is mediated by a mechanism that is distinct from mechanisms of T cell suppression previously identified in schistosome-infected mice, including anergy induction.

### T cell suppression induced by pre-patent schistosome infection is cell contact-dependent

**Figure 7:**
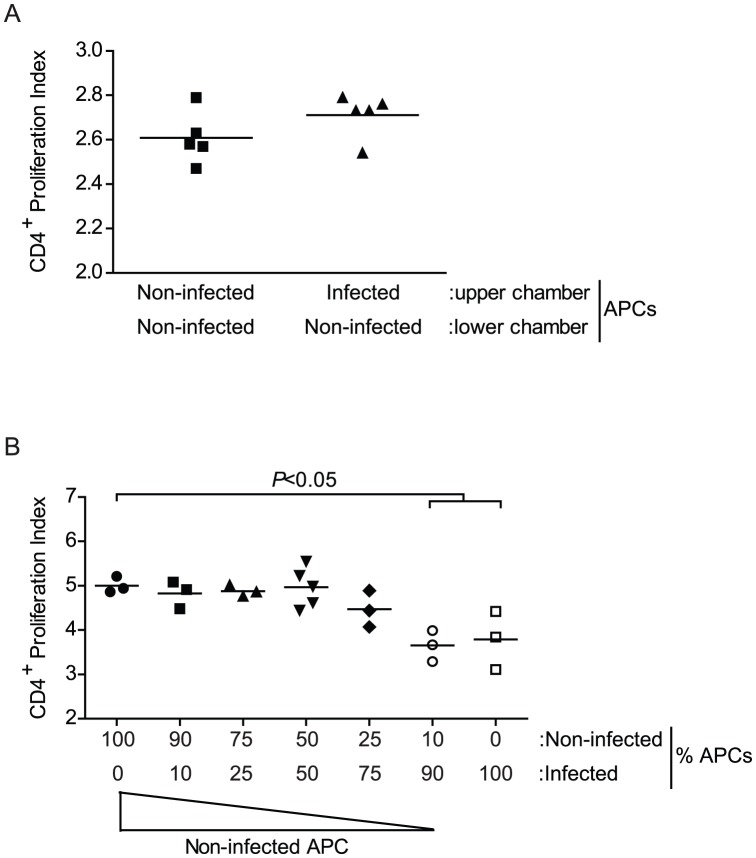
T cell suppression induced by pre-patent schistosome infection is cell contact-dependent. A, Transwell experiment was performed where the lower chamber contained splenocytes isolated from non-infected RAG-1^−/−^ mice co-cultured with CFSE-stained CD4^+^ T cells isolated from non-infected OT-II/RAG-1^−/−^ mice. Upper chamber contained splenocytes isolated from 4 week *S. mansoni*-infected or non-infected RAG-1^−/−^ mice. Cells were co-cultured for 72 hours in the presence of OVA peptide and CD4^+^ T cell proliferation was analyzed by flow cytometry. Horizontal bars represent mean of 5 independent mice. B, Competition assay was performed where varying proportions of splenocytes isolated from 4 week *S. mansoni*-infected or non-infected RAG-1^−/−^ mice were co-cultured with CFSE stained CD4^+^ T cells isolated from non-infected OT-II/RAG-1^−/−^ mice. CD4^+^ T cell proliferation was analyzed by flow cytometry. Horizontal bars represent mean of 5 independent mice. APCs, antigen presenting cell.

As our data failed to support a role for several established soluble mediators in limiting T cell responsiveness, we next tested whether the mechanism of T cell suppression was mediated by a soluble, diffusible mediator or required cell contact, using cell-impermeable Transwell inserts. The lower chamber contained splenocytes from non-infected RAG-1^−/−^ mice plus OVA peptide and CFSE-labeled OT-II T cells. Splenocytes from either infected or non-infected RAG-1 ^−/−^ mice were then added to the upper chamber and the proliferation of the T cells in the lower chamber was assessed following three days of culture. Regardless of whether the upper chamber contained splenocytes from infected or non-infected RAG-1^−/−^ mice, there was no difference in the proliferation of the OT-II T cells in the lower chamber (Fig. 7A), suggesting that the suppression of T cell proliferation by splenocytes from infected mice is not caused by the production of a diffusible inhibitor.

To test whether splenocytes from infected RAG-1^−/−^ mice could inhibit the stimulatory capacity of splenocytes from non-infected RAG-1^−/−^ mice, we performed competition assays, where splenocytes from infected and non-infected RAG-1^−/−^ mice were mixed in varying proportions and used as APCs to stimulate OT-II T cells. When splenocytes from infected mice comprised 10–75% of the APCs, there was no significant difference in proliferation of the T cells when compared to T cells cultured in the presence of non-infected RAG-1^−/−^ splenocytes alone (Fig. 7B). Indeed, no significant impairment in T cell proliferation was detected until the infected RAG-1^−/−^ splenocytes outnumbered those from non-infected mice by a ratio of 9∶1, when proliferation levels were similar to those obtained with infected RAG-1^−/−^ splenocytes alone (Fig. 7B). Together, these data suggest that T cell suppression by splenocytes from infected mice is not mediated by a soluble factor, but instead requires cell-to-cell contact. Furthermore, these data suggest that, when compared cell-for-cell, splenocytes from infected mice cannot suppress the T cell proliferation stimulated by splenocytes from non-infected mice until they outnumber cells from non-infected mice by a ratio of more than 3∶1.

### Suppression of IL-12 expression may contribute to impairment of T cell responses during pre-patent schistosome infection

**Figure 8:**
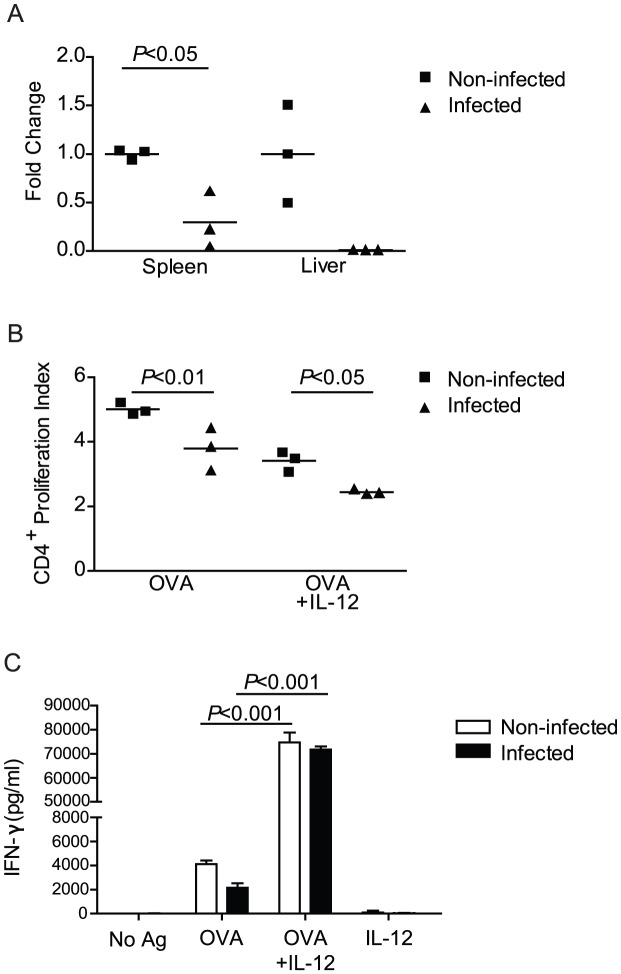
Suppression of IL-12 expression may contribute to impairment of T cell responses during pre-patent schistosome infection. A, Taqman real-time PCR gene expression assay for IL-12p35 was performed on RNA isolated from tissue of the spleen or liver of 4 week *S. mansoni*-infected or non-infected mice wild type mice. Fold change was calculated using the comparative C_T_ method and GAPDH as the endogenous control. B and C, Splenocytes isolated from 4 week *S. mansoni* infected or non-infected RAG-1^−/−^ mice or and CFSE-stained CD4^+^ T cells isolated from non-infected OT-II/RAG-1^−/−^ mice were co-cultured for 72 hours without antigen, with indicated combinations of OVA peptide and IL-12, or with anti-CD3 antibody. CD4^+^ T cell proliferation was analyzed by flow cytometry (B) and the concentration of IFN-γ in the cell culture supernatants was measured by ELISA (C). Horizontal bars represent mean of 3 independent mice (A, B). Data shown are mean +/− SEM (C). No Ag, no antigen; OVA, OVA peptide; αCD3, anti-CD3 antibody.

As T cell suppression by splenocytes from infected RAG-1^−/−^ mice required cell contact and did not occur unless splenocytes from infected mice outnumbered those from non-infected mice, we hypothesized that the T cell suppression resulted from a loss in T cell stimulatory capacity rather than production of a suppressive mediator. This hypothesis is also consistent with our finding that splenocytes from infected mice only inhibited T cell proliferation when they outnumbered splenocytes from non-infected mice, because it is only under these conditions that APCs from infected mice would out-compete competent APCs for interactions with T cells. Proliferation of T cells and their production of IFN-γ is potently stimulated by IL-12, a T cell stimulatory factor produced by CD11b^+^ and CD11c^+^ APCs. We therefore hypothesized that defective T cell stimulation by APCs from infected mice may be due to a failure to produce adequate IL-12. Consistent with this hypothesis, we found that pre-patent schistosome infection resulted in significant down-regulation of IL-12p35 mRNA expression in the spleen by 4 weeks post infection (Fig. 8A). A similar trend was observed in liver tissue, although the difference was not statistically significant. As exogenous IL-12 has been shown to reverse anergy and restore T cell production of IFN-γ [49], [50], we tested whether addition of IL-12 to co-cultures containing infected APCs could restore cytokine production and T cell proliferation. Adding exogenous IL-12 did not restore proliferation and in fact appeared to further impair proliferation of OT-II T cells, regardless of whether APCs from infected or non-infected mice were used (Fig. 8B). However, addition of IL-12 dramatically increased IFN-γ production by OVA peptide-stimulated OT-II cells when splenocytes from either infected or non-infect mice (Fig. 8C) were used. Indeed, when IL-12 was included in the cultures, there was no longer any difference in IFN-γ production, regardless of whether APCs from infected and non-infected mice were used. These data suggest that IL-12 down-regulation in innate APCs during pre-patent infection contributes to impaired T cell cytokine production. However, there are likely other factors that contribute to the mechanism of T cell suppression, as IL-12 did not restore T cell proliferation.

## Discussion

Helminth infections are classically associated with the induction of Th2 and regulatory responses, the latter resulting in diminished immunoresponsiveness to both helminth [27], [51], [52] and bystander antigens [53]. We previously showed that during pre-patent schistosome infection, migration of juvenile worms from the skin to the portal vasculature is associated with systemic induction of antigen-specific CD4^+^ T cells that produce IFN-γ, IL-4 and IL-10 in response to schistosome worm antigens [8]. Here we provide evidence that induction of schistosome-specific CD4^+^ T cell responses by pre-patent infection is accompanied by loss of CD4^+^ T cell responsiveness to non-schistosome antigens. These results are similar to previously published findings that showed a reduction in T cell responsiveness to polyclonal stimulation during patent schistosome infection [31]. However, to our knowledge, our data are the first to suggest a similar loss of T cell responsiveness occurs before the onset of egg-laying.

Schistosome infection induces regulatory T cells that play a significant role in controlling Th responses and modulating the development of egg-induced pathology [54], [55]. However, our demonstration that T cell hyporesponsiveness also affects OVA-specific T cells in OT-II/RAG-1^−/−^ mice, which are devoid of all other T cells, indicates that T cell hyporesponsiveness can occur independent of Treg and other adaptive responses. Rather, our data suggest that loss of T cell responsiveness in this instance occurs as a result of direct alterations in innate immune function. Confirming the role of innate cells in mediating T cell hyporesponsiveness, the loss of T cell cytokine production and proliferation was recapitulated when OT-II T cells from non-infected mice were stimulated with splenocytes from infected RAG-1^−/−^ mice. These results argue that T cell hyporesponsiveness does not occur as a result of some infection-induced cell-intrinsic change in T cell function. Indeed, we have previously shown that isolated OT-II CD4^+^ T cells respond equally well to OVA or anti-CD3 stimulation, regardless of whether they are isolated from infected or non-infected OT-II/RAG-1^−/−^ mice [56]. Therefore we conclude from these data that pre-patent schistosome infection induces changes in innate cells that impair their ability to stimulate CD4^+^ T cell responses.

Professional APCs of the innate immune system consist of two main populations belonging to the mononuclear phagocyte system, namely dendritic cells and monocytes/macrophages. In particular, dendritic cells are known to play a vital role in the immune response to schistosomes and several reports highlight the importance of CD11c^+^ dendritic cells in priming Th2 cell responses during schistosome infection [57], [58], [59], [60]. Liver macrophages have also been implicated in inducing the activation and type 2 differentiation of CD4^+^ T cells during schistosome infection [61]. CD4^+^ T cell stimulation by APCs requires expression of MHC class II-peptide complexes and co-stimulatory ligands [62], [63], [64] and reduced expression of these molecules by APCs during T cell priming can cause T cells to become unresponsive [48]. However, our data did not provide any evidence that pre-patent infection impaired the expression of MHC class II or CD86 by either APC population, or diminished their numbers in either liver or spleen. In contrast, when CD11c^+^ and CD11b^+^ cells were isolated from infected mice and their ability to stimulate CD4^+^ T cells in vitro was compared, we found that a significant defect in T cell stimulation was associated with the CD11b^+^ cells and not the CD11c^+^ cells. These observations suggest that CD11c^+^ dendritic cells are functionally intact and that impairment of DC function is not the cause of reduced T cell responsiveness during pre-patent infection. Rather, our findings suggest that impairment of CD11b^+^ APC function is responsible, at least in part, for the loss of T cell responsiveness seen during pre-patent infection. These findings are consistent with previous evidence supporting a regulatory role for macrophages during schistosome infection. For example, a schistosome polysaccharide with immunomodulatory properties was found to mediate its effects by eliciting a population of suppressor macrophages that suppressed CD4^+^ T cell proliferation [23]. In another study, macrophages from infected mice were shown to be responsible for the induction of T cell anergy during schistosome infection [44].

In these previous studies, the mechanisms implicated in the suppression of T cell proliferation by macrophages were the production of NO [23] and the expression of PD-L1 (B7-H1) [44]. In contrast, our data suggest that neither of these mechanisms accounts for the suppression of T cell responses in our system. Minor differences in the methods employed may account for some of the discrepancies between our results and those previously reported, including our use of schistosome infection rather than glycoconjugate exposure [23], and our use of naïve monospecific T cells and peptide antigen (OVA) rather than anti-CD3 stimulation of polyclonal T cell populations with presumably mixed naïve, activated and memory phenotypes [44]. However, there are other mechanisms by which CD11b^+^ cells may suppress T cell proliferation. For example arginase, which together with nitric oxide synthase participates in arginine metabolism, has been implicated in the suppression of T cell proliferation by myeloid-derived suppressor cells [45]. Furthermore, the regulatory cytokine IL-10 is produced by both T cell and non-T cell sources during schistosome infection [54], and has been shown to inhibit the priming of Th1 responses by dendritic cells [55]. Finally, TGF-β, a regulatory cytokine produced by macrophages and other cells, was shown to suppress T cell responses in schistosome infection [65]. However, interference with each of these other regulatory pathways also failed to restore T cell proliferation, suggesting that the mechanism responsible for T cell suppression we observed is distinct from mechanisms of macrophage-mediated T cell suppression and anergy induction previously identified in schistosome infection. Further underlying this distinction, we found that provision of exogenous IL-2, which has been shown to reverse clonal anergy in CD4^+^ T cells [47], also did not restore T cell proliferation in the presence of splenocytes from infected mice, suggesting the T cells are not subject to anergy induction.

As we could find no evidence that any of these candidate mechanisms were significantly influencing APC function, we questioned whether the observed impairment was cell contact-dependent or -independent and whether infection causes innate APCs to produce a diffusible suppressive or inhibitory molecule. Consistent with data suggesting that the T cell suppression we observed was not mediated by the diffusible factors IL-10 and TGF-β, we found evidence that the T cell suppression was contact-dependent, as no impairment of T cell proliferation was observed unless the T cells were mixed directly with splenocytes from infected mice in the same well. Furthermore, T cell proliferation was not significantly impaired until splenocytes from infected mice outnumbered those from non-infected mice by a ratio of 3∶1. These findings suggest the “suppressor” cells induced by infection are not potent suppressors of T cells. Rather, these cells may simply be impaired in their capacity to stimulate T cells, such that T cell suppression only occurs when they are present in sufficient numbers to out-compete competent APCs. In support of a model whereby APC stimulatory capacity is lost rather than an active suppressor mechanism induced, we found that pre-patent schistosome infection was associated with a significant decrease in the baseline expression of IL-12, a critical stimulator of T cell proliferation and IFN-γ production. Indeed, addition of exogenous IL-12 to T cell-splenocyte co-cultures stimulated robust IFN-γ production, even when splenocytes from infected mice were used as APCs. Consistent with a potential role for IL-12 downregulation in T cell suppression, previous studies showed that schistosome egg antigens suppressed IL-12 production by LPS-stimulated dendritic cells [66]. Furthermore, a recent study demonstrated that IL-12 production was suppressed in the intestines of mice harboring patent schistosome infections, by a mechanism that requires arginase I expression [67]. However, schistosome-induced impairment of IL-12 expression is likely not the only factor that contributes to loss of T cell responsiveness during pre-patent infection, as exogenous IL-12 did not restore T cell proliferation in T cell-splenocyte co-cultures. Thus, we propose a model where pre-patent schistosome infection leads to a loss of T cell stimulatory capacity amongst innate accessory cells, and therefore to T cell suppression, by a mechanism that involves, but is not limited to, inhibition of IL-12 expression.

In summary, we have shown that CD11b^+^ cells exposed to pre-patent schistosome infection are impaired in their ability to stimulate CD4^+^ T cells and that this may contribute to an overall reduction of T cell responsiveness during pre-patent infection. Furthermore, we have provided evidence to suggest that infection-induced impairment of IL-12 production contributes to this loss of T cell stimulatory capacity. Future studies will seek to identify other aspects of T cell stimulation that are suppressed by pre-patent schistosome infection and will examine the mechanisms by which juvenile schistosomes induce these suppressive effects. An understanding of the host-parasite interactions that lead to immune hyporesponsiveness may identify opportunities to selectively stimulate immunity against helminths in infected and susceptible hosts.
